# Status and perspectives of hospital mortality in a public urban Hellenic hospital, based on a five-year review

**DOI:** 10.1186/1471-2458-8-28

**Published:** 2008-01-23

**Authors:** Iordanis N Papadopoulos, Maria Papaefthymiou, Leonidas Roumeliotis, Vasilios G Panagopoulos, Anna Stefanidou, Anastasia Kostaki

**Affiliations:** 1 National University of Athens, 'Attikon' University General Hospital, Fourth Surgery Department, 1 Rimini Street, 124 62, Athens, Greece; 2 Department of Medical Informatics, General Hospital of Nikaias Piraeus, 3 Maduvalou Street, 18454 Nikaias, Piraeus, Greece; 3 Athens University of Economics and Business, Department of Statistics, 76 Patission Avenue, 104 34 Athens, Greece

## Abstract

**Background:**

Analysis of hospital mortality helps to assess the standards of health-care delivery.

**Methods:**

This is a retrospective cohort study evaluating the causes of deaths which occurred during the years 1995–1999 in a single hospital. The causes of death were classified according to the International Statistical Classification of Diseases (ICD-10).

**Results:**

Of the 149,896 patients who were discharged the 5836 (3.4%) died. Males constituted 55% and females 45%. The median age was 75.1 years (1 day – 100 years).

The seven most common ICD-10 chapters IX, II, IV, XI, XX, X, XIV included 92% of the total 5836 deaths.

The most common contributors of non-neoplasmatic causes of death were cerebrovascular diseases (I60–I69) at 15.8%, ischemic heart disease (I20–I25) at 10.3%, cardiac failure (I50.0–I50.9) at 7.9%, diseases of the digestive system (K00–K93) at 6.7%, diabetes mellitus (E10–E14) at 6.6%, external causes of morbidity and mortality (V01–Y98) at 6.2%, renal failure (N17–N19) at 4.5%, influenza and pneumonia (J10–J18) at 4.1% and certain infectious and parasitic diseases (A00–B99) at 3.2%, accounting for 65.3% of the total 5836 deaths.

Neoplasms (C00–D48) caused 17.7% (n = 1027) of the total 5836 deaths, with leading forms being the malignant neoplasms of bronchus and lung (C34) at 3.5% and the malignant neoplasms of large intestine (C18–21.2) at 1.5%. The highest death rates occurred in the intensive care unit (23.3%), general medicine (10.7%), cardiology (6.5%) and nephrology (5.5%).

Key problems related to certification of death were identified. Nearly half of the deaths (49.3%: n = 2879) occurred by the completion of the third day, which indicates the time limits for investigation and treatment. On the other hand, 6% (n = 356) died between the 29^th ^and 262^nd ^days after admission.

Inadequacies of the emergency care service, infection control, medical oncology, rehabilitation, chronic and terminal care facilities, as well as lack of regional targets for reducing mortality related to diabetes, recruitment of organ donors, provision for the aging population and lack of prevention programs were substantiated.

**Conclusion:**

Several important issues were raised. Disease specific characteristics, as well as functional and infrastructural inadequacies were identified and provided evidence for defining priorities and strategies for improving the standards of care. Effective transformation can promise better prospects.

## Background

Health care demands are rapidly expanding in Greece and upgrading of the health care system is a pressing need. Priorities for transformation are not always easy to define and should be based on evidence. Analysis of hospital mortality can help to assess the standards of health care delivery. Within this context this study investigated the mortality in a single urban, public hospital, the General Hospital of Nikaias Piraeus. The hospital accommodates 750 beds and accepts patients for acute and elective, secondary and tertiary, medical and surgical care. The hospital belongs to the Third Regional Health and Welfare System of Greater Athens, which serves a population of approximately 1,500,000 and is constituted by eight national hospitals, five health care centers, and fourteen regional infirmaries. These offer in total approximately 4,200 available beds and employ approximately 7,500 professionals of various specializations [1].

## Methods

### Aims, design and settings

The objectives of this retrospective cohort study were to substantiate the status and to identify disease-specific, functional and infrastructural factors associated with the hospital mortality, in order to produce evidence for upgrading health care.

For this purpose, the causes of the in-hospital deaths among those who were treated and discharged from the General Hospital of Nikaias Piraeus, during the five years starting on 1 January 1995 and ending on 31 December 1999 were evaluated.

In Greece, there is a legal obligation for a medical professional to certify death, to complete the international form of death certificate, which is recommended by the World Health Organization (WHO) [2], and to refer the deceased for autopsy and death certification when the cause of death is not clear.

### Data source and measurements

The data of this study were extracted from the electronic database of the hospital and included demographics, medical specialty involved in the care of the patients, dates of admission and discharge and the causes and dates of deaths as were recorded in the electronic and un-coded forms of death certificates.

The causes of death were coded according to the International Statistical Classification of Diseases and related health problems, 10^th ^Revision (ICD-10) [3]. When more than one cause of death was stated in Part I of the death certificate, the proper sequence of the underlying causes was selected. The underlying cause of death was taken as defined in the ICD-10, i.e. the disease or injury that initiated the train of morbid events leading directly to death, or the circumstances of the accident or violence that produced the fatal injury.

Death certificates were a source of structured data available in electronic form. However, focusing and classifying deaths by the underlying cause results in the underestimation of diseases that are extremely important for assessing the workload and the status of the hospital care when the intention is to produce evidence for upgrading health care. For this reason additional analyses with respect to relative frequencies of common diseases for which the patients were actually treated in the hospital such as, diabetes mellitus, hypertensive diseases, cardiac failure, renal failure, acute tubulo-interstitial nephritis, influenza and pneumonia as well as sepsis, septicemia and septic shock were performed.

In order to evaluate consistency in coding, the first three authors worked together in coding the first 100 death certificates. Consequently, the second and third authors classified all the causes of death independently and the intra-rater variation was tested.

### Statistics

Kappa statistics were used to identify consistency between the two coders [4]. All statistical analyses were performed using the Statistical Package for the Social Sciences version 10^th ^(SPSS, Chicago, Illinois, USA). The ethics committee of the General Hospital of Nikaias Piraeus, Greece, has approved this study.

## Results

### Sample size

Over the five-year period studied, 5836 patients of those who were admitted in this hospital died. The deceased represent 3.4% of the 149,896 patients who were discharged from the hospital and 1.9% of the 303,366 who had a planned outpatient consultation.

During the same period 850,000 patients had a consultation in the accident and emergency department. The sample size of the study represents 1.15% of all the 506,608 deaths that occurred throughout Greece during the same time and are reported by the National Statistics Service.

### Gender and age

Males constituted 55% (n = 3208) and females 45% (n = 2628) of the total 5836 deceased. The median age for males and females was 75.1 years (ranged: less than 1 day to 100 years), but 73.1 (ranged: less than 1 day to 99 years) for the male and 77.3 (ranged: less than 1 day to 100 years) for the female subsets.

As to the ages at death, 3725 (63.7%) of the deceased were over 70 years old, while 1000 (17.1%) of the deceased were 85 years or older. The gender and age distribution are shown in Table 1.

**Table 1:** Gender and age distributions of the 5836 deceased.

Age groups	Males and females, n	Males and females, %	Males, n	Females, n
0–4	84	1.4	47	37
5–9	2	0.0	1	1
10–14	3	0.1	2	1
15–19	16	0.3	12	4
20–24	34	0.6	26	8
25–29	40	0.7	23	17
30–34	36	0.6	20	16
35–39	54	0.9	37	17
40–44	67	1.1	41	26
45–49	116	2.0	78	38
50–54	175	3.0	129	46
55–59	181	3.1	114	67
60–64	414	7.1	264	150
65–69	663	11.4	425	238
70–74	884	15.1	499	385
74–79	900	15.4	484	416
80–84	941	16.1	453	488
>85	1000	17.1	430	570
Known age	5610	96.1	3085	2525
Missing data	226	3.9	123	103
Total	5836	100	3208	2628

### Death rates per medical specialty

A subset of 2775 (47.5% of the total 5836) deaths occurred while the patients were under the care of general medicine physicians, 1148 (19.7%) under cardiologists, 496 (8.5%) under general surgeons and 384 (6.6%) under neurosurgeons, accounting for 4803 (82.3%) of the total 5836 deaths. The highest death rates (deaths over discharged from specializing unit) occurred in the intensive care unit (23.3%), the general medicine (10.7%), cardiology (6.5%) and nephrology (5.5%). The distribution of death rates per specialty is shown in Table 2.

**Table 2:** Distribution of death rates per specialty.

Specialty	Deaths, n	% of deaths	Discharged patients, (All years)
General medicine	2775	47,5	25975
Cardiology	1148	19,7	17697
General surgery	496	8,5	16413
Neurosurgery	384	6,6	8759
Chest physicians	218	3,7	4318
Nephrology	192	3,3	3494
Orthopedics	153	2,6	7368
Intensive care unit	120	2,1	514
Neurology	87	1,5	4438
Neonatology	80	1,4	1507
Urology	64	1,1	10141
Thoracic surgery	55	0,9	1841
Remaining specialties	51	0,9	47431
Unknown	13	0,2	
Total	5,836	100	149,896

### Hospitalization time

The median hospitalization time was three days (ranged from less than 24 hours to 262 days) and 75% and 95% of the deaths occurred by the 9^th ^and 31^st ^days from admission, respectively. On the other hand, 6% (n = 365) died in the hospital between 29 and 262 days from admission. The distribution of deaths from the time of admission is shown in Table 3.

**Table 3:** The distribution of deaths from the time of admission.

Day	Frequency, n	Per cent*	Cumulative percentage
< 1	811	13,9	13,9
1	1039	17,8	31,7
2	574	9,8	41,5
3	455	7,8	49,3
4	359	6,2	55,5
5	307	5,3	60,8
6	234	4	64,8
7	224	3,8	68,6
2^nd ^week	962	16,5	85,1
3^rd ^week	328	5,5	90,7
4th week	186	3,2	93,9
Day 29 to 262	356	6	100
Total	5835		
Missing	1		

*From day 18 to 31 the percentage ranged from 0.3 to 0.7%.

### Consistency in coding the causes of death

A high consistency was found between the two coders in coding the causes of death, with 5526 of the 5836 receiving identical codes (k = 0.943, p = 0.003). A subset of 310 deaths initially classified in different chapters by the two authors. The most common differences between the two coders observed in the following blocks: A00–B99, certain infectious and parasitic diseases (n = 14, 0.3%); C00–D48, neoplasms: all codes (n = 15, 0.4%); C00–D48, neoplasms (all codes except the following specified) (n = 12, 0.2%); E00–E90, endocrine, nutritional and metabolic diseases: all codes (n = 10, 0.2%); E10–E14, diabetes mellitus (n = 11, 0.2%); I00–I99, diseases of the circulatory system (all codes except the following specified) (n = 14, 0.3%); I50.0–I50.9, cardiac failure (n = 11, 0.2%); J00–J99, diseases of the respiratory system: all codes (n = 27, 0.4); J10–J18, influenza and pneumonia (n = 17, 0.3%); K00–K93, diseases of the digestive system (n = 17, 0.3%); V01–Y98, external causes of morbidity and mortality: all codes (n = 17, 0.3%). Consequently the 310 deaths were reviewed by the three first authors, consensus was achieved and the resulted classification was further analyzed.

### Relative frequencies of common causes of death

The following seven most common ICD-10 chapters IX (Diseases of the circulatory system: all codes), II (Neoplasms: all codes), IV (Endocrine, nutritional and metabolic diseases: all codes), XI (Diseases of the digestive system), XX (External causes of morbidity and mortality: all codes), X (Diseases of the respiratory system: all codes), and XIV (Diseases of the genitourinary system: all codes) included 92% (n = 5369) of the total 5836 deaths and are shown in Table 4.

**Table 4:** Classification of the causes of death according to ICD-10.

ICD-10 Chapter	Blocks	ICD-10 Title	No of deceased	*Per cent of total 5836 deceased
I	A00–B99	Certain infectious and parasitic diseases	185	3.2
II	C00–D48	Neoplasms: all codes	1027	17.7
	C00–D48	Neoplasms (all codes except the following specified)	351	6.0
	C16–16.9	Malignant neoplasms of stomach	62	1.1
	C18–21.2	Malignant neoplasms of large intestine	88	1.5
	C34	Malignant neoplasms of bronchus and lungs	203	3.5
	C50	Malignant neoplasms of the breast	47	0.8
	C51, C52, C54, C55, C57, C58	Malignant neoplasms, female genital organs (coded)	12	0.2
	C53	Malignant neoplasms of the uterine cervix	6	0.1
	C56	Malignant neoplasms of the ovary	17	0.3
	C61	Malignant neoplasms of the prostate	33	0.6
	C64–68	Malignant neoplasms of the urinary tract	74	1.3
	C69–72	Malignant neoplasms of eye, brain, and other parts of central nervous system	56	1.0
	C81–C96	Malignant neoplasms, of the lymphoid, haemopoietic and related tissue	49	0.8
	D46	Myelodysplastic syndrome	29	0.5
III	D50–D89	Diseases of the blood and blood-forming organs and certain disorders involving the immune mechanism	42	0.7
IV	E00–E90	Endocrine, nutritional and metabolic diseases: all codes	437	7.5
	E00–E90	Endocrine, nutritional and metabolic diseases (all codes except the following specified)	51	0.9
	E10–E14	Diabetes mellitus	384	6.6
	E65–E68	Obesity and other hyperalimentation	2	0.0
V	F00–F99	Mental and behavioral disorders	6	0.1
VI	G00–G99	Diseases of the nervous system	40	0.7
IX	I00–I99	Diseases of the circulatory system: all codes	2461	42.1
	I00–I99	Diseases of the circulatory system (all codes except the following specified)	313	5.4
	I10–I15	Hypertensive diseases	99	1.7
	I20–I25	Ischemic heart diseases	603	10.3
	I46	Cardiac arrest	61	1.0
	I50.0–I50.9	Cardiac failure	462	7.9
	I60–I69	Cerebrovascular diseases	923	15.8
X	J00–J99	Diseases of the respiratory system: all codes	348	6.0
	J00–J99	Diseases of the respiratory system (all codes except the following specified)	21	0.4
	J10–J18	Influenza and pneumonia	240	4.1
	J40–J47	Chronic lower respiratory diseases	69	1.2
	J96	Respiratory failure not classified elsewhere	18	0.3
XI	K00–K93	Diseases of the digestive system	393	6.7
XII	L00–L99	Diseases of the skin and subcutaneous tissue	16	0.3
XIII	M00–M99	Diseases of the musculoskeletal system and connective tissue	14	0.2
XIV	N00–N99	Diseases of the genitourinary system: all codes	341	5.8
	N00–N99	Diseases of the genitourinary system (all codes except the following specified)	13	0.2
	N10–N16	Acute tubulo-interstitial nephritis	67	1.1
	N17–N19	Renal failure	261	4.5
XVI	P00–P96	Certain conditions originating in the perinatal period	65	1.1
XVII	Q00–Q99	Congenital malformations, deformations and chromosomal abnormalities	18	0.3
XVIII	R00–R99	Symptoms, signs and abnormal clinical and laboratory findings, not elsewhere classified	7	0.1
XX	V01–Y98	External causes of morbidity and mortality: all codes	362	6.2
	X60–X84	Intentional self-harm	2	0.0
		Not classifiable**	74	1.3
Total			5836	100.0

*Per cent of the total 5836 deceased in hospital.

**These deceased were referred for autopsy, the death certificates were issued directly by the forensic medical department and the database of the hospital was not updated.

There were no recorded deaths in the following ICD-10 blocks; diseases of the eye and adnexa (H00–H59), diseases of the ear and mastoid process (H60–H95), pregnancy, childbirth and the puerperium (O00–O99), complications of medical and surgical care (Y40–Y84), factors influencing health status and contact with health services (Z00–Z99) and under the codes for special purposes (U00–U99).

### Non-neoplasmatic causes of death

The most common contributors of non-neoplasmatic causes of death were cerebrovascular diseases (I60–I69) at 15.8%, ischemic heart disease (I20–I25) at 10.3%, cardiac failure (I50.0–I50.9) at 7.9%, diseases of the digestive system (K00–K93) at 6.7%, diabetes mellitus (E10–E14) at 6.6%, external causes of morbidity and mortality (V01–Y98) at 6.2%, renal failure (N17–N19) at 4.5%, influenza and pneumonia (J10–J18) at 4.1% and certain infectious and parasitic diseases (A00–B99) at 3.2%. These most common causes together accounted for 65.3% of the total 5836 deaths.

### Common diseases within the ICD-10 chapters

With respect to the relative frequencies of common diseases within the ICD-10 chapters, the following was revealed. Sepsis, septicemia or septic shock (non-distinguishably used) was the most commonly reported cause of death of the 185 patients classified in certain infectious and parasitic diseases (A00–B99), accounting for 66.4% (n = 123). Influenza and pneumonia (J10–J18) caused 69% (n = 240) of the 348 deaths due to diseases of the respiratory system (J00–J99). Renal failure (N17–N19) was the commonest cause of death, with 76.5% (n = 261), and acute tubulo-interstitial nephritis (N10–N16) the second most common cause of death, with 19.6% (n = 67), of the total of 341 deaths attributed to diseases of the genitourinary system (N00–N99). Of the 437 deaths classified in the 'endocrine, nutritional and metabolic diseases' (E00–E90), diabetes mellitus (E10–E14) caused 87.8% (n = 384). Coding diabetes mellitus (E10–E14) as underlying cause of death included 76 patients who were treated with cardiac failure and 45 patients treated with renal failure. Coding hypertensive diseases (I10–I15) as underlying cause of death also included 18 patients treated for cardiac failure and 1 patient treated for renal failure. Hence, the actual workload of patients with cardiac failure was 556 (9.5% of the total 5836) and the sum of patients with renal failure was 307 (5.2% of the total 5836).

### Neoplasms

Neoplasms (C00–D48) caused 17.7% (n = 1027) of the total of 5836 deaths, with leading forms being malignant neoplasms of bronchus and lung (C34) at 3.5%, malignant neoplasms of the large intestine (C18–21.2) at 1.5%, malignant neoplasms of the urinary tract (C64–68) with 1.3%, malignant neoplasms of the stomach (C16–16.9) with 1.1%, and malignant neoplasms of the breast (C50) with 0.8%. The complete distribution of the causes of death per ICD-10 chapter is shown in Table 4.

### Gender in relation to causes of death

Overall, the deaths of males exceeded the deaths of females by 10%. 'Certain infectious and parasitic diseases' (A00–B99) were 1.2 times more common in males, at a rate 1.8% (n = 104) versus 1.4% (n = 81) in females. Neoplasms (C00–D48) were 1.6 times more common in males, at a rate 10.8% (n = 633) versus 6.8% (n = 394) in females. Malignant neoplasms of bronchus and lungs (C34), of the urinary tract (C64–68) and of the stomach (C16–16.9) were 6, 3.3, and 2.2 times more common in males than in females, respectively. Diseases of the circulatory system (I00–I99) were 1.1 times more common in males, at a rate of 22.5% (n = 1313) versus 19.7% (n = 1148) in females. Diseases of the respiratory system (J00–J99) were 1.3 times more common causes of death in males, at a rate 3.4% (n = 199) versus 2.6% (n = 149) in females.

Diseases of the digestive system (K00–K93) were 1.3 times more common in males, at a rate 3.8% (n = 222) versus 2.9% (n = 171) in females. External causes of morbidity and mortality (V01–Y98) were 1.5 times more common causes of death in males, at a rate 3.8% (n = 227) versus 2.4% (n = 135) in females.

A notably increased rate of deaths in females was observed with respect to endocrine, nutritional and metabolic diseases (E00–E90), at a rate 4.3% (n = 252) versus 3.2% (n = 185) and diabetes mellitus (E10–E14) was 1.3 times more common in females.

### Age in relation to causes of death

The most common causes of death for those aged 15–39 years were external causes of morbidity and mortality (V01–Y98). For those aged over 40 years, and throughout the age range, cerebrovascular diseases (I60–I69) was the most common cause of death, and for those aged over 75 years, the rates of cerebrovascular diseases (I60–I69), ischemic heart diseases (I20–I25) and cardiac failure (I50.0–I50.9) were the predominant causes of death. Deaths due to neoplasms (C00–D48) reached the peak of incidence at the ages 65–79, while deaths due to endocrine, nutritional and metabolic diseases (E00–E90) reached their peak at the ages 70–74 years.

**Figure 1:**
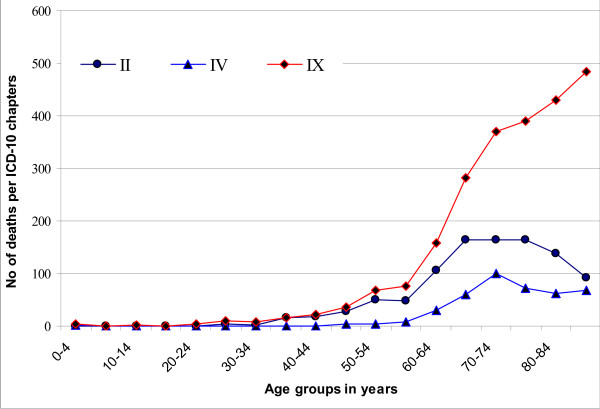
**Distribution of deaths of the three most common ICD-10 chapters over the age groups**. Distribution of 3791 deaths in the following three chapters; IX: Diseases of the circulatory system (I00–I99, n = 2368); II: Neoplasms (C00–D48, n = 1004); IV: Endocrine, nutritional and metabolic diseases (E00–E90, n = 419).

**Figure 2:**
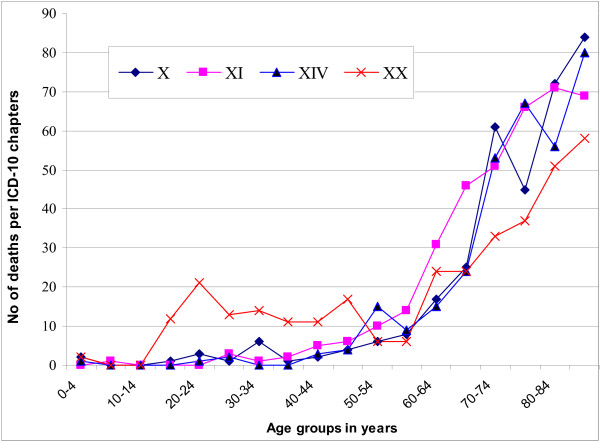
**Distribution of deaths of the fourth, fifth, sixth and seventh most common ICD-10 chapters, over the age groups**. Distribution of 1384 deaths included in the following four chapters; XI: Diseases of the digestive system (K00–K93, n = 376); XX: External causes of morbidity and mortality (V01–Y98, n = 340); X: Diseases of the respiratory system (J00–J99, n = 338); XIV: Diseases of the genitourinary system (N00–N99, n = 330).

The distribution of 5175 deaths of the seven most common ICD-10 chapters over the age-groups included (96.3%) of the total 5369 deaths of these chapters because for the remaining the ages were missing (Figure 1 and Figure 2).

### Hospitalization time in relation to gender, age and cause of death

Before the completion of the first 24 hours after admission, ischemic heart diseases (I20–I25), cerebrovascular diseases (I60–I69) and heart failure (I50–I50.9) were the first, second and third most common causes of deaths, respectively.

Cerebrovascular diseases (I60–I69) were the predominant causes of death in the period from 24 hours until 15 days after admission. Death rates due to ischemic heart diseases (I20–I25) as well as those due to external causes of morbidity and mortality (V01–Y98) reached their peaks before the completion of the first 24 hours.

Neoplasms (C00–D48) were the third most common causes of death from the 8^th ^to the 23^rd ^day, became the first most common cause between the 24^th ^and 30^th ^day, and declined to the second most common cause, following cerebrovascular diseases (I60–I69), after one month.

### Autopsy rates

The annual rates of autopsies for those who where admitted and treated in the hospital varied from 7.1 to 9.9%, hence only this proportion of death certificates was based on autopsy findings. The dead on arrival to hospital subjects and those who died in the accident and emergency department before the admission to the wards were not recorded in the electronic database of the hospital and were not further evaluated. Manual research of the archive of 5 years showed that 905 of the 2466 who were dead on arrival or died in the emergency department referred for autopsy and the corresponding annual autopsy rates varied from 34.5% to 40.8%.

### Limitations on data collection

Proportional hospital mortality was used in this study because access to all hospitals of the country is open to the whole of the population, and estimation of population based data was difficult. In addition, the total 149,896 discharge-diagnoses of this hospital although available in an electronic form they were not coded by the ICD-10. This study was focused on the in-hospital mortality, but the one-month post-discharge mortality rate of those who left the hospital alive was not known.

The date of birth was not recorded in the case of 226 (3.9%) of the deceased, and inadequacy of the data precluded the classification of the causes of death of 74 (1.3%). The latter occurred because the deceased was referred for autopsy, hence the death certificates were issued directly by the forensic medical department but the database of the hospital was not updated. For a total of 61 (1.0%) of the deaths, cardiac arrest (I46) was the only recorded diagnosis in Part I and II of the death certificates.

## Discussion

### Problems related to death certification and proposed actions

This study evaluated the mortality at a single urban hospital which accommodates only 17.8% of the total beds of this health care region. Completeness of death certificates with respect to date of birth (missing in 3.9%), and missing data in describing the causes of death (not classifiable for 1.3%) could be increased by vigilance in data collection and processing. However, inaccuracies associated with the identification of the causes of death are commonly reported and are related to diagnostic errors [5], to errors in completing the death certificates [6], as well as to errors with respect to the coding process.

In this hospital, the annual autopsy rate (7.1 to 9.9%) for those who receive in-hospital care should be increased if it is going to be used for systematic audit, while the high autopsy rate (34.5% to 40.8%) of those who were dead on arrival or died in the accident and emergency department is a subject of further research that may reveal useful information. Substantial improvement of the infrastructure is required to meet the needs of systematic audit by necropsy.

Training, certification of the death by the senior caring physician, and the increasing use of necropsy rates, have been proposed in order to improve the accuracy of data [6,7]. Furthermore, the work of international agencies such as WHO [3], EUROSTAT [8], and the National Center for Health Statistics of the USA [9] with the International Collaborative Effort on the consistency, comparability and automation of the cause of death coding systems, should minimize biases in coding and increase the accuracy of mortality data.

In the present study, the absence of recordings with respect to complications of medical and surgical care (Y40–Y84) should be further investigated. Reassurance of the profession from the fear of litigation should yield more accurate data for evaluation of the standards of care.

Currently, coding is centralized by the National Statistics Service but the feedback to the hospital-based clinicians is poor. Trained nosological coders may monitor the mortality, co-ordinate the process of certification and encourage regular and comparative reviews with the medical records [10].

### Gender and age

National data confirm a general pattern of decreasing mortality rates from 1967 to 1996 [11] and a life expectancy at birth (in 2004) of 77 years for males and 82 years for females [12]. The excess by 10% of males among the deceased in this study indicates that a larger proportion of females died at home. Males were more prone to suffer the most common causes of death, with the exception of the endocrine, nutritional and metabolic diseases (E00–E90).

### Causes of death: Can the trends be reversed?

Proportional hospital mortality which was used in this study has its limitations. Certain infections and parasitic diseases (A00–B99) caused 185 (3.2%) of the deaths of this study, and sepsis, septicemia and septic shock (non-distinguishably measured) was the most commonly reported cause of death at 66.4% (n = 123). The overall prevalence of hospital-acquired infections reported in 14 out of 112 Greek hospitals was 9.3% [13], but it is not known for this hospital. The recently established at a national level infection control groups should provide the clinicians with such important data and organize effective surveillance.

Diseases of the circulatory system (I00–I99) caused 2461 (42.1%) of the deaths in this study. The reported burden of the total cardiovascular mortality in the European countries represents around 40% of all-causes mortality for the ages 45–74 years [14]. Cerebrovascular diseases (I60–I69) were the leading causes of death in the present study, with 923 (15.8%) deceased. The reported 28-day case fatality rate from Greece was 26.6% [15]. Stroke related mortality has declined in Europe over the period 1970–1996, but not in Greece.

Ischemic heart disease (I20–I25) was the second most common cause of death, accounting for 603 (10.3%) of the deceased of this study. The age-adjusted mortality due to ischemic heart disease in Greece increased from the 1960s to the 1990s [16,17], while it is declining in the USA [18].

In this study, cardiac failure (I50.0–I50.9) caused a high proportion of deaths (n = 462, 7.9%). However, it was observed that the ICD-10 coding underestimated the workload of the hospital with respect to cardiac failure and the actual sum of patients with cardiac failure was 556 (9.5% of the total 5836). Cardiac failure is a major public health problem with a reported in-hospital mortality of 4% [19].

The findings of the present study indicate that further investigation is required with respect to early diagnosis and the standards of management of the diseases of the circulatory system particularly with respect to cardiac failure, in order to reverse the current trends of mortality.

Neoplasms (C00–D48) caused 1027 deaths (17.7%) of this study. An increasing trend of mortality due to neoplasms throughout the period 1967–1996 has been recently reported in Greece [11] while the overall number of cancer deaths observed in the European Union recently has reduced by 10% [20].

Malignant neoplasms of bronchus and lung (C34) was the dominant neoplasm in this study, causing 203 (3.5%) of the deaths. As has been reported in the United States, lung cancer coded as an underlying cause of death in death certificates captured almost 92% of all the deceased diagnosed with lung cancer [21].

Malignant neoplasms of the large intestine (C18–21.2) caused 88 (1.5%) of all the deaths in this study, being the second most common cause of deaths due to noeplasms. Malignant neoplasms of the large intestine are reported as the second most common cause of deaths due to neoplasms in Europe also [22]. Malignant neoplasms of the breast (C50) caused 47 (0.8%) of the deaths in this study. The reported age-standardized mortality rate due to breast cancer in Greece is 15.9% [23]. The risk of dying from breast cancer in the European Union fell by 5% over the recent years, but an increase of 7% has been observed in Greece [20].

Malignant neoplasms of the stomach (C16–16.9) caused 62 (1.1%) of the deaths of this study. The risk of dying from gastric cancer in the European Union is declining, but the trend is unknown in this region [20].

Malignant neoplasms of the prostate (C61) caused 33 (0.6%) of the deaths of this study. In the European Union, the risk of dying from cancer of the prostate rose by 5% in 2000 [20].

Despite the fact that approximately one-fifth of the total deaths of the study were due to neoplasms, there are no medical oncology departments in this hospital, and no established screening programs for the asymptomatic population in the region.

Diabetes mellitus (E10–E14) was the underlying cause of 384 (6.6%) deaths in this study. People with diabetes mellitus have a substantially reduced life expectancy, a twofold higher risk of death from cardiovascular disease when compared to non-diabetics [24] and a high risk of developing renal failure. Discrepancies in the coding practice for diabetes mortality have been shown in European countries [8,25].

The data of this study substantiate the need for specification of regional targets for reducing mortality related to diabetes, as has been previously recommended [26].

Diseases of the respiratory system (J00–J99) caused 348 (6%) of the deaths of this study. Influenza and pneumonia (J10–J18) caused 240 (4.1%) of the deaths. Lower respiratory tract infection has been reported recently as the leading cause of mortality among hospitalized patients with hospital-acquired infections in 14 Greek hospitals [27]. The high proportion of deaths attributed to diseases of the genitourinary system (N00–N99) in this study (n = 341: 5.8%), is due to a large number of patients with chronic renal failure (N17–N19: n = 261: 4.5%). This is due to a high volume of patients being treated in a specialized unit of this hospital. An additional number of patients were classified under the codes of diabetes mellitus (E10–E14) and hypertensive diseases (I10–I15) and the actual sum of patients with renal failure was 307 (5.2% of the total 5836). Effective regional diabetes control prevention programs, as well as increasing the rates of kidney transplantation by recruiting organ donors from trauma-victims offers hope for these patients.

Certain conditions originating in the perinatal period (P00–P96) consisted of 65 (1.1%) of the total deaths, mainly due to premature deliveries. Maternal mortality in Greece has significantly decreased over the last few decades [28].

External causes of morbidity and mortality (V01–Y98) caused 362 (6.2%) of the deaths of the present study and were the most common cause of death for people under 39 years. Preventable trauma deaths and the need for regionalization of trauma care have been substantiated by recent studies from this region [29,30]. With respect to mortality associated with natural disasters, potentially preventable deaths from the 1999 Athens earthquake, which affected this region, have recently been reported [31].

Intentional self-harm (X60–X84) is a largely preventable public health problem [32]. The reported suicide mortality rate in Greece over the last decade was approximately 2.8 per 100,000 [33,34]. These low rates have been attributed to the Greek life style, and the characteristic strong family ties. However, the low rate which was observed in this study is misleading, because the deceased are often referred directly to the forensic medical departments for issuing the death certificate and not to the hospitals. Effective prevention is likely to remain elusive, in the absence of adequate data on suicide epidemiology [34].

### Hospitalization time, acute and chronic care facilities

This study has shown that nearly half (n = 2879, 49.3%) of the total deaths had occurred by the completion of three days from admission, a figure that indicates the time-limits for in-hospital investigation and treatment. Ischemic heart diseases (I20–I25) and external causes of morbidity and mortality (V01–Y98) reached their peaks before the completion of the first 24 hours, hence effective urgent treatment of these groups of patients is vital. On the other hand, 6% (n = 356) of the patients died in the hospital between the 29^th ^and 262^nd ^days after admission, amongst whom cerebrovascular diseases (I60–I69) and neoplasms (C00–D48) were the first and second most common causes of death, respectively. This long time period reflects the lack of rehabilitation as well as lack of chronic health care facilities in the region. While 1000 (17.1%) of the deceased were 85 years or older, there are no geriatric wards in this hospital.

### Clinical audit

The essence of this study was to audit health care delivery and despite its limitations it raised and substantiated many relevant and important issues. The apparent high rates of mortality in some specialties, and particularly that of general medicine (10.7%), is partly due to the fact that they accommodate many patients for terminal care. The data of this study do not allow further stratification of severity of the diseases. The nature, as well as, the timing of the received therapeutic interventions were unknown, hence further conclusions with respect to standards of care cannot be drawn. Nowadays, it is essential for the medical profession to become the pioneers in establishing regular and effective clinical audit and this study should help in this process.

### Study implications, priorities and perspectives

The problems related to the process of death certification should be promptly resolved. The high volume of admissions and emergency room consultations, in association with the time-limits for investigation and treatment of patients with acute and severe diseases, imply that there is a need for urgent investment and regionalization of the emergency in-hospital care, the pre-hospital emergency medical care and the primary health care sectors of this region.

Infection control, particularly with respect to hospital-acquired infections; the establishment of medical oncology as well as rehabilitation, chronic and terminal health care facilities; regional targets for reducing mortality related to diabetes; recruitment of organ donors and provision for the aging population are some of the issues that need prompt solutions.

Certain well-known risk factors of our lifestyle, such as tobacco [35], and particularly those related to diseases of the circulatory system, to neoplasms and to the respiratory system, are avoidable [15,16]. Public education, trauma prevention, and establishment of screening programs in the region are of vital importance for long term health care benefits. This study shows that there is a place for improving the standards of care, and such effective interventions promise better future perspectives.

## Conclusion

Problems relating to death certification were identified. Monitoring mortality and clinical audit should help to improve the standards of care. Disease specific characteristics were identified and priorities with respect to functional integration and upgrading the infrastructure were suggested. This study should help in planning the reduction of mortality rates. Upgrading of the system promises better prospects in health care delivery and for the longevity of the population of this region.

## Competing interests

The author(s) declare that they have no competing interests.

## Authors' contributions

Concept and design of the study: INP

Data extraction and management: VGP, AS, AK

Coding causes of death: MP, LR, INP, VGP

Statistical analysis: AK, AS, INP

Drafting, writing the paper and interpretation: INP, LR, MP.

All authors have read and approved the final version of the manuscript.

## Pre-publication history

The pre-publication history for this paper can be accessed here:


